# Probing the plasmonic near-field by one- and two-photon excited surface enhanced Raman scattering

**DOI:** 10.3762/bjnano.4.94

**Published:** 2013-12-02

**Authors:** Katrin Kneipp, Harald Kneipp

**Affiliations:** 1 Physics Department, Technical University of Denmark, 2800 Kgs Lyngby, Denmark

**Keywords:** near-field, plasmonics, silver nanoaggregates, single molecule, surface-enhanced Raman scattering (SERS)

## Abstract

Strongly enhanced and spatially confined near-fields in the vicinity of plasmonic nanostructures open up exciting new capabilities for photon-driven processes and particularly also for optical spectroscopy. Surface enhanced Raman signatures of single molecules can provide us with important information about the optical near-field. We discuss one- and two-photon excited surface enhanced Raman scattering at the level of single molecules as a tool for probing the plasmonic near-field of silver nanoaggregates. The experiments reveal enhancement factors of local fields in the hottest hot spots of the near-field and their dependence on the photon energy. Also, the number of the hottest spots and their approximate geometrical size are found. Near-field amplitudes in the hottest spots can be enhanced by three orders of magnitudes. Nanoaggregates of 100 nm dimensions provide one hot spot on this highest enhancement level where the enhancement is confined within less than 1nm dimension. The near-field enhancement in the hottest spots increases with decreasing photon energy.

## Introduction

The resonance frequencies of collective oscillations of the electrons in the conduction band in metal nanostructures, which are called surface plasmons, fall in the optical range of the electromagnetic spectrum. This results in a strong coupling between incident light and surface plasmons. The interaction gives rise not only to beautifully colored glass windows in old cathedrals but also to strongly enhanced and spatially highly confined local fields in the vicinity of such plasmonic structures [1–2]. Exploiting these optical near-fields opens up exciting new capabilities for photon-driven processes and particularly for optical spectroscopy. Surface-enhanced Raman scattering (SERS) might be one of the most prominent effects to demonstrate the potential of spectroscopy performed in the near-field. SERS enables Raman measurements on a single molecule [3]. Vice versa, here we show that single molecules and their Raman signatures can be useful tools for probing plasmonic near-fields.

The measurement of Raman signals from single molecules requires high optical field intensities in order to compensate for the extremely small Raman cross sections. Enhancement of the near-field intensity is the key effect in surface enhanced Raman scattering. High local fields are generated by a redistribution of field intensities in the vicinity of a plasmonic nanostructure. This results in a wide intensity distribution within the near-field. Sites that provide extremely high local fields, so-called “hot spots” exist along with areas showing much lower level of field intensity. Theory predicts the highest local fields in gaps between silver and gold nanostructures, e.g., in aggregates of nanoparticle of various sizes and shapes reaching from dimers [4–7], and trimers [8] to selfsimilar structures formed by silver- or gold nanospheres [9]. High local fields can also exist in fractal films or cavities of these noble metals [10]. The recently reported super-resolution imaging of SERS on silver nanoaggregates has directly confirmed nanoparticle junctions to be responsible for single molecule SERS [11].

Computations show dramatic variations in near-field intensities within a few nanometers. Even sophisticated optical experiments cannot reveal these dramatic spatial variations. However, surface plasmons can be also excited by low energy [12] and high energy electrons [13–14]. Therefore, as an alternative to optical methods, electron energy loss spectroscopy (EELS) is emerging as a novel tool to probe plasmonic near-fields of metal nanostructures at nanometer-resolutions and even below [15–19]. We have applied EELS for probing the local distribution of plasmonic fields at nanometer scale for nanoaggregates formed by silver particles [18].

Here we discuss experiments for probing the optical near-field of silver nanoaggregates by using one- and two-photon excited Raman scattering. The applied excitation wavelengths do not match the electronic transitions of the target molecules, i.e., SERS spectra are measured without additional contributions of an intrinsic molecular resonance Raman enhancement. The experiments are performed in a way that single molecules reside exclusively in the hottest hot spots provided in the near-field. We employ surface-enhanced pumped anti-Stokes Raman scattering (SEPARS) and one- and two-photon excited surface enhanced Raman (SERS) and hyper Raman (SEHRS) signals, respectively for estimating the maximum field enhancement in these hot spots. Measuring SERS signals over wide concentration ranges of a target molecule displays the step-by-step occupation of the near-field volume by Raman active molecules and enables to infer a geometrical size of the hottest hot spots as well as their number.

## Results and Discussion

### Plasmonic silver nanoaggregates

**Figure 1:**
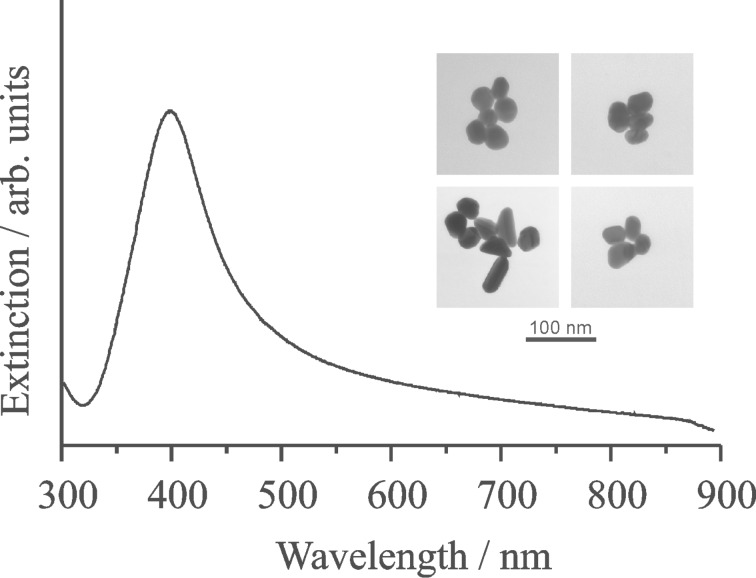
Electron micrographs of typical silver nanoaggregates that were used in this study and the extinction spectrum of their aqueous solution.

The highest SERS enhancement levels obtained so far are related to silver or gold nanoparticles that form aggregates as “SERS-active substrates” and by using excitation in the near infrared [20–22]. The first SERS spectra of single molecules had been measured by using small aggregates of silver nanoparticles and still, such aggregates seem to be the favorite “plasmonic substrate” for single-molecule SERS [23]. Most single-molecule SERS studies are using excitation wavelengths that are resonant with electronic transitions in the target molecule and, by this way, exploit a superposition of enhanced local fields and resonance Raman scattering (SERRS) [7,23–28]. These resonant conditions, and even pre-resonant conditions, greatly reduce the requirements for local field intensities since these experiments take advantage of the large cross section for the molecular Raman resonance [29–30]. However, exploiting extremely high local fields also enables Raman measurements from single molecules without the support of an intrinsic molecular resonance Raman enhancement [29,31–36]. Figure 1 shows typical silver nanoaggregates that provide extremely high near-field enhancement levels, which are suitable for single-molecule SERS under non-resonant conditions. The silver structures were prepared by a standard citrate reduction procedure [37].

In this study, SERS experiments are carried out in solutions of silver nanoaggregates under the condition that the concentration of the target molecules is by a factor of 10–100 smaller than the concentrations of the plasmonic nanoaggregates. This means that, statistically, single nanoaggregates carry a single target molecule, while most of the aggregates are “empty”. Single-molecule SERS experiments have shown that it is likely that these single molecules reside in the hottest hot spots of the nanoaggregates [29]. This is also supported by computations that show that high field gradients related to hot spots in plasmonic near-fields might direct single molecules to the hottest spots [38]. Experiments performed under these conditions exclusively probe the hottest spots of the near-fields in an ensemble of plasmonic nanoaggregates.

### Probing the field enhancement in the hottest hot spots of the near-field

As we discussed above, single molecules can be subnanometer-size sensors for probing the near-field of nanoaggregates. Here we infer the enhancement factor for the near-field in the hottest hot spots by considering the anti-Stokes to Stokes signal ratios during surface-enhanced pumped anti-Stokes Raman scattering (SEPARS) experiments as well as the ratios between two- and one-photon excited surface enhanced hyper Raman and Raman scattering SEHRS and SERS, respectively.

#### Surface-enhanced pumped anti-Stokes Raman scattering(SEPARS)

**Figure 2:**
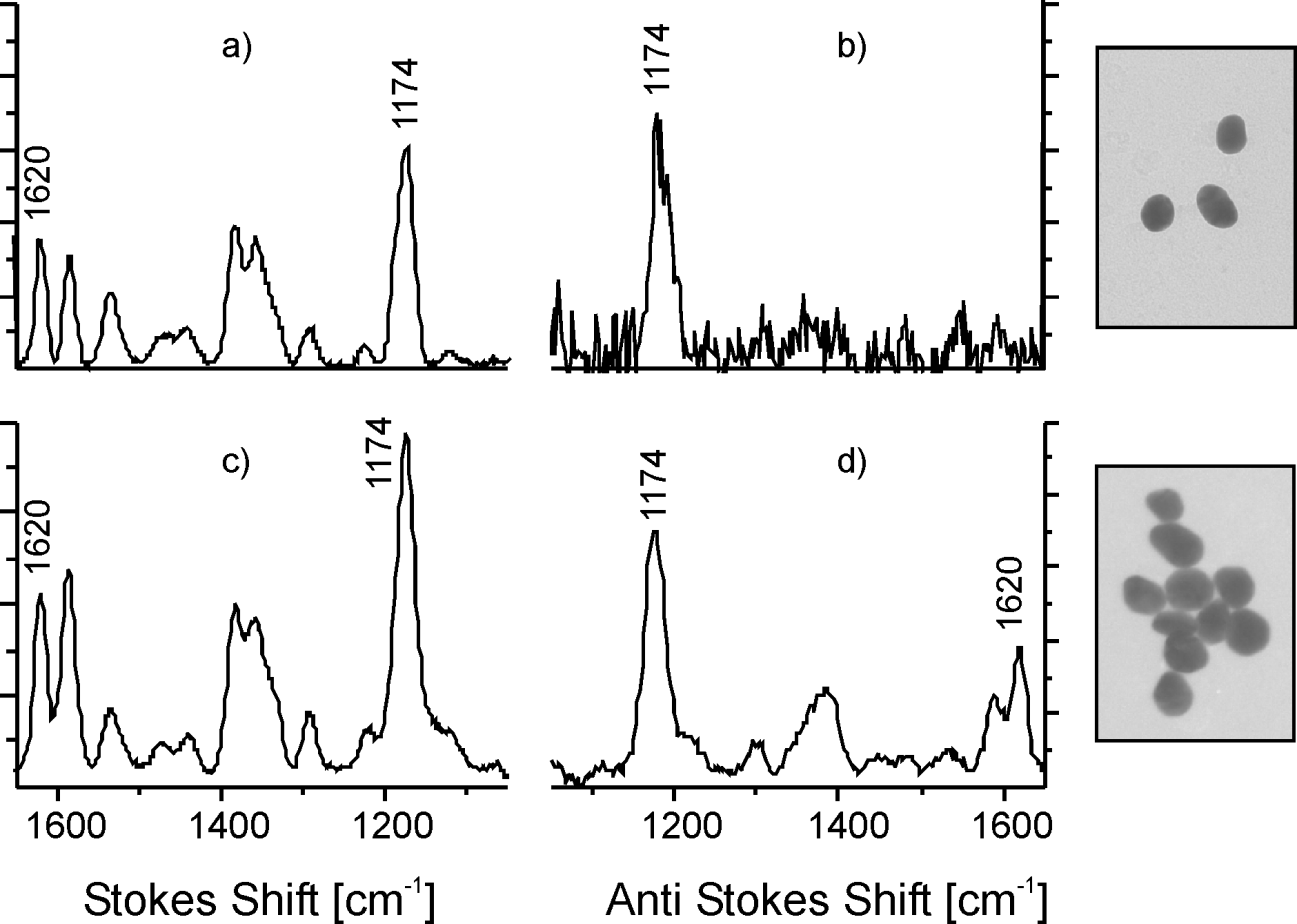
Stokes and anti-Stokes SERS spectra of crystal violet under conditions of “normal” SERS (a,b) and extremely strong SERS (c,d) [31]. Spectra were measured by using 830 nm non-resonant excitation. Reprinted with permission from [22], Copyright 2006 American Chemical Society.

While Stokes Raman scattering starts from the vibrational ground states of a molecule, anti-Stokes Raman scattering is related to scattering with molecules in the first excited vibrational states. Therefore, the ratio between anti-Stokes and Stokes Raman signals is determined by the ratio of the number of molecules *N*_1_ and *N*_0_ in these two states, i.e., by the Boltzmann population. This results in much weaker anti-Stokes Raman signals than Stokes signals, particularly for higher frequency vibrational modes. However, the situation can be dramatically changed in a very strong surface-enhanced Raman process. This is illustrated in Figure 2: Isolated silver or gold nanoparticles support a surface-enhanced Raman signal of crystal violet shown in the Stokes spectrum (Figure 2a). As expected, the anti-Stokes SERS spectrum (Figure 2b) displays the lower frequency modes only. This situation changes when aggregates are employed as enhancing structures for spectra (Figure 2c) and Figure 2d). Now a strong anti-Stokes spectrum appears.

**Figure 3:**
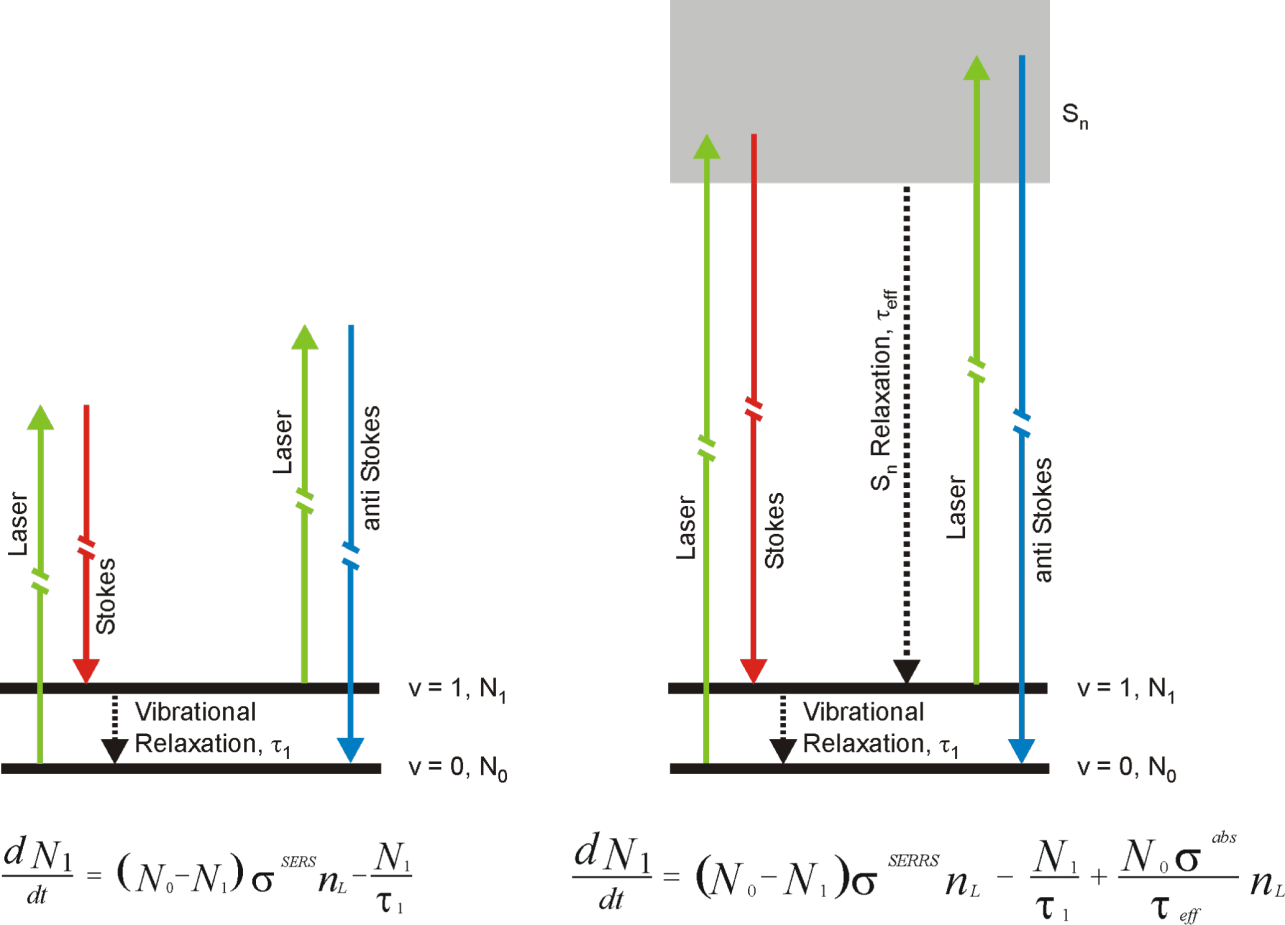
Non-resonant (left) and resonant (right) Stokes and anti-Stokes Raman scattering in an energy level diagram. The rate equations describe the population of the first excited vibrational level. *N*_1_ and *N*_0_ are the numbers of molecules in the excited and ground vibrational state, respectively. σ^SERS^ and σ^SERRS^ are effective SERS and SERRS cross sections, respectively, σ^abs^ describes electronic absorption. τ_1_ is the lifetime of the first excited vibrational state, τ_eff_ the inverse rate constant for the population of the first excited vibrational level by internal conversion followed by vibrational relaxation and/or by fluorescence. *n*_L_ is the number of laser photons per cm^2^ and second applied for the excitation.

In particular, this spectrum shows strong anti-Stokes lines also for the higher-energy Raman modes. The strong anti-Stokes SERS signal is related to the effect of surface enhanced pumped anti-Stokes Raman scattering (SEPARS). During SEPARS, Stokes scattering with an extremely high effective cross section populates the first excited vibrational levels in addition to the thermal population [20,33,39]. This results in an increase of anti-Stokes signals. In Figure 2c and Figure 2d, SEPARS can be observed due to highly localized and enhanced fields of silver nanoaggregates, which result in extremely high effective SERS cross sections. This is in contrast to the relatively weakly enhanced local fields for isolated silver particles that were used in Figure 2a and Figure 2b. Figure 3 displays the energy level diagram for non-resonant and resonant Stokes and anti-Stokes Raman scattering along with rate equations that describe the population of the first excited vibrational level.

While for non-resonant Raman scattering, a strong Stokes process appears to be the only way to “pump” vibrational levels, in case of resonant and pre-resonant Raman scattering, other mechanism(s) such as ultrafast internal conversion after electronic excitation and/or fluorescence exist as main source for the population of excited vibrational states. This has been confirmed by time resolved pump–probe anti-Stokes SERS studies on rhodamine 6G on silver nanostructures with an excitation wavelength of 633 nm [40]. These experiments identified a rise time of the pumped anti-Stokes signal of about 0.8 ps related to the population of the excited vibrational level by ultrafast internal conversion. Population pumping due to a Raman process follows the excitation pulse instantaneously. Therefore, for the extraction of information on effective SERS cross sections, SERS enhancement factors and enhanced near-fields, population pumping should be employed under non-resonant Raman conditions. Here we discuss SEPARS by using non-resonant excitation in the NIR wavelengths range. Since the ratio between anti-Stokes and Stokes Raman signals is determined by the ratio between *N*_1_ and *N*_0_, a simple equation for the anti-Stokes to Stokes signal ratio *P*_aS_*/P*_S_ can be derived from the left rate equation in Figure 3 by assuming steady state and weak saturation [20]

[1]
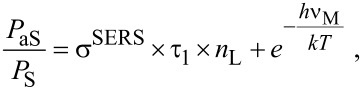


where ν_M_ is the molecular vibrational frequency, *T* is the temperature of the sample, and *h* and *k* are the Planck and Boltzmann constant, respectively. The first term in Equation 1 describes the anti-Stokes to Stokes signal ratio related to SERS vibrational pumping due to a strong Raman Stokes process. In “normal” non-resonant and also in resonant Raman scattering with cross sections on the order of 10^−30^ to 10^−24^ cm^2^, this term can be neglected since it is small compared to the thermal population described by the second term. In order to account for experimentally observed anti-Stokes to Stokes signal ratios in SERS experiments on silver nanoaggregates, as it is demonstrated for example in Figure 2c and Figure 2d, the product of cross section and vibrational lifetime in Equations 1 must be on the order of 10^−27^ cm^2^·s. With vibrational lifetimes on the order of 10 ps [40], effective cross sections that can account for Raman pumping of a molecular vibration have to be at least on the order of 10^−16^ cm^2^.

In general, an effective SERS cross section can be written as [39]

[2]
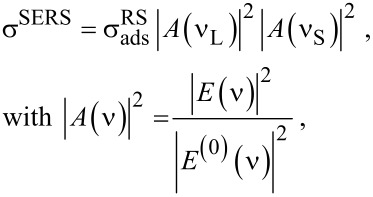


where *A*(ν) describes the enhancement of the near-field at the location of the molecule, *E*(ν) is the local optical field (laser and the scattered field, respectively) and *E*^(0)^(ν) is the field in the absence of the metal nanostructure. σ^RS^_ads_ is the Raman cross section of a molecule in contact with the metal compared to σ^RS^_free_ of a molecule without coupling to the metal. The ratio σ^RS^_ads_/σ^RS^_free_ describes the enhancement effects related to “chemical” or “first layer” mechanisms in SERS [41–42].

SERS vibrational pumping based on effective Raman cross sections on the order of 10^−16^ cm^2^ has been also observed for adenine attached to silver nanoaggregates [33]. The molecule absorbs in the UV, i.e., the applied excitation photons at 830 nm are at a much lower energy than the electronic transitions and SERS on adenine cannot benefit from a molecular-resonance Raman effect. Therefore, effective SERS cross sections on order of 10^−16^ cm^2^ compared to typical non-resonant Raman cross sections on the order of 10^−30^ cm^2^ imply SERS enhancement factors of around 10^14^. Anti-Stokes to Stokes signal ratios measured on different structures from 100 nm aggregates to larger fractal silver structures that were several micrometers in size show that enhancement factors on the order of 10^14^ in non-resonant SERS are independent of the size and shape of the aggregates. However, they critically depend on the gap size between the nanoparticles forming the aggregate [22,33,43]. In general, enhanced local fields *and* “chemical” enhancement can contribute to such enormous non-resonant total enhancement factors. SEPARS experiments provide us with information about the total effective SERS cross section (see Equation 2). In the following section, we discuss hyper Raman scattering as a method to separate information about the near-field enhancement and for probing local fields in hot spots in more detail.

#### Surface enhanced two-photon excited hyper Raman scattering

**Figure 4:**
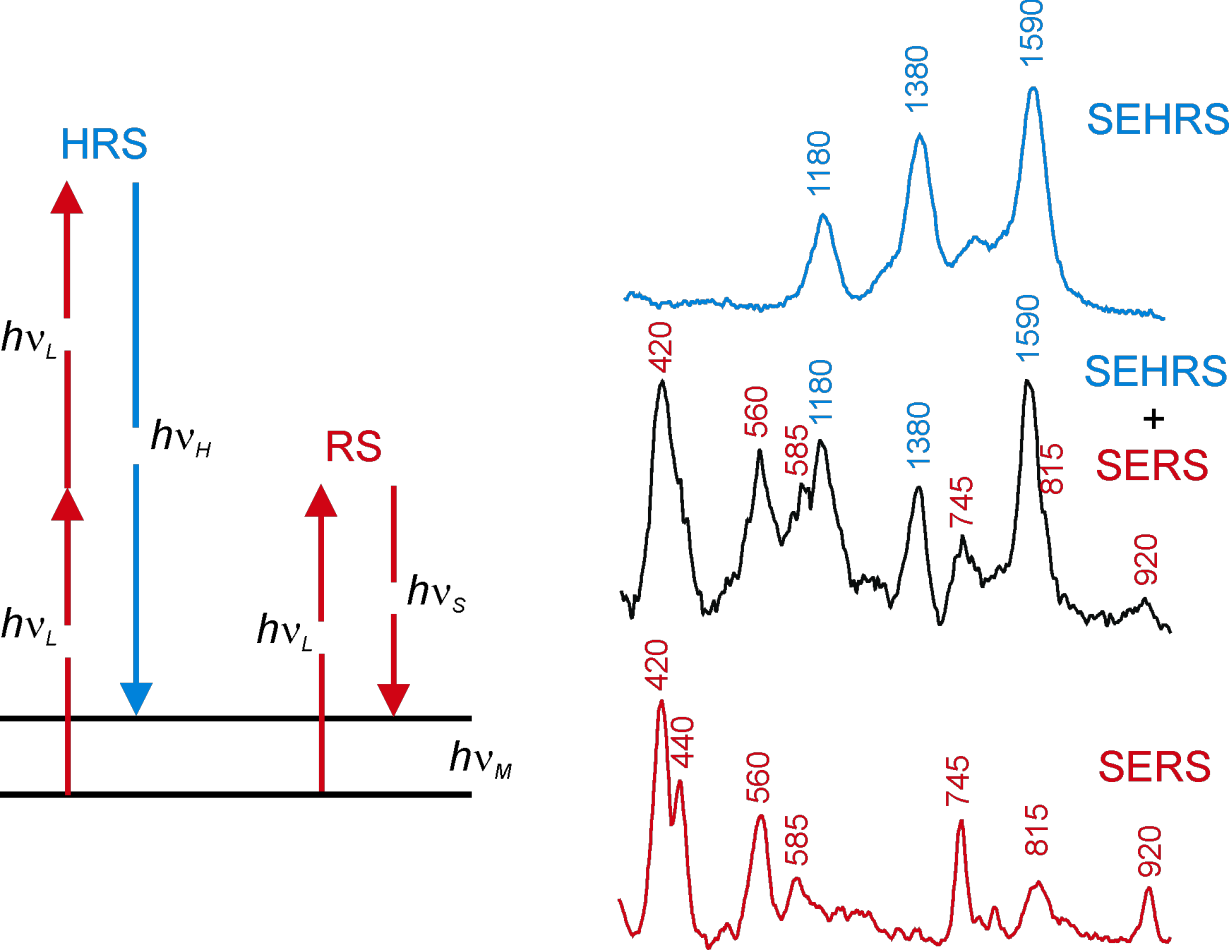
Schematic of hyper Raman- and Raman scattering (left) and surface enhanced Raman- and hyper Raman spectra of crystal violet on silver nanoaggregates (right, excitation 850 nm, 10^7^ W/cm^2^)_._ SEHRS and SERS signals of crystal violet measured in the same spectrum by simultaneously using the first and second diffraction order of the spectrograph (middle trace). In the upper and the lower trace, SEHRS and SERS spectra are differentiated by placing a NIR absorbing filter in front of the spectrograph or by switching off the mode locked regime of the Ti:sapphire laser, respectively [44]. Reprinted with permission from [22], Copyright 2006 American Chemical Society.

During hyper Raman scattering (HRS), two photons interact simultaneously with the molecules. This process results in an incoherent scattering signal shifted relative to the second harmonic of the excitation laser. Figure 4 explains hyper Raman scattering in an energy level diagram. HRS follows symmetry selection rules that are different from those in Raman scattering, and therefore it can probe vibrations that are forbidden in Raman scattering.

As it is generated by two-photon excitation, the HRS signal is a quadratic function of the excitation intensity. Extremely small cross sections on the order of 10^−65^ cm^4^s/photon make the utilization of HRS as a practical spectroscopic tool nearly impossible. However, HRS particularly benefits from optical near-fields. The effective cross section for surface enhanced hyper Raman scattering (SEHRS) can be written as

[3]
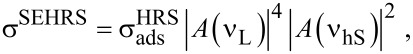


where σ^HRS^_ads_ describes a “chemically” enhanced hyper Raman cross section compared to that of a “free” molecule, *A*(ν) describes the enhancement of the near-fields at the excitation and hyper Raman scattered wavelengths, respectively.

**Figure 5:**
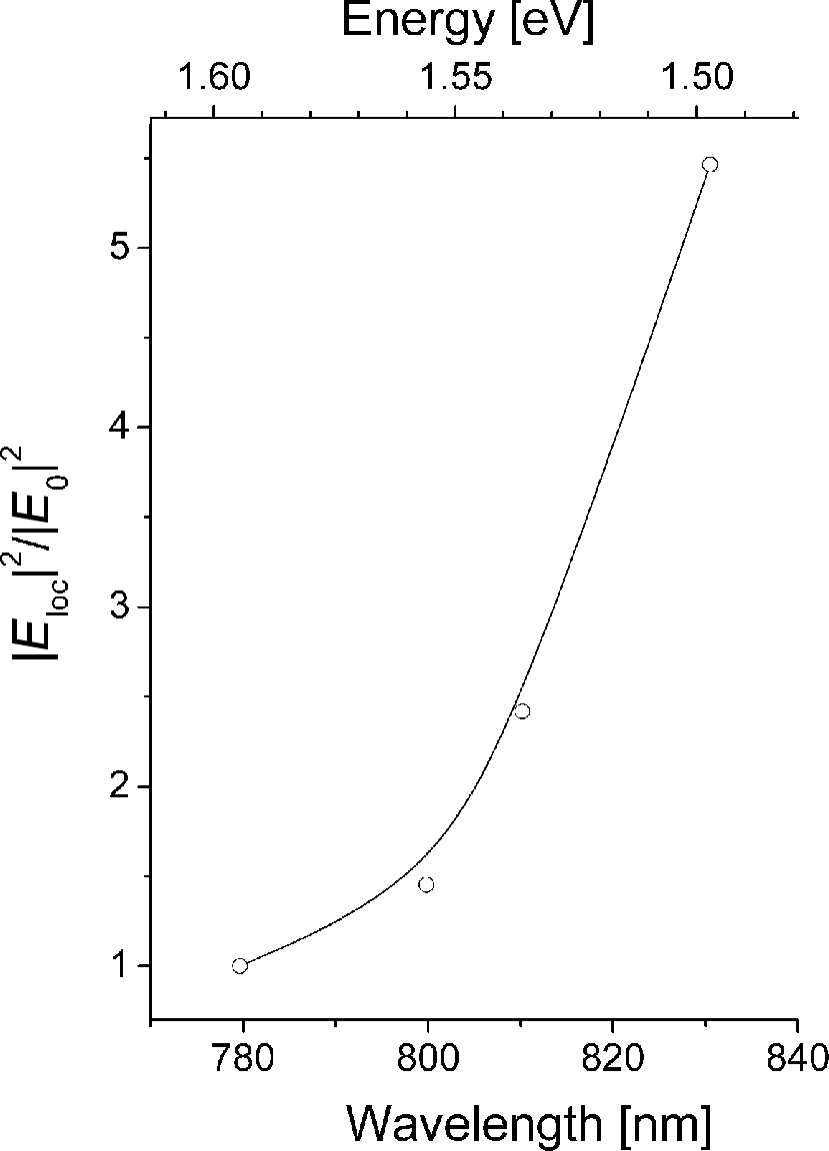
Near-field intensity *E*_loc_^2^/*E*_0_^2^ in the hot spots inferred from the ratio of SEHRS and SERS signals measured for crystal violet on silver nanoaggregates while using excitation wavelengths between 780 and 830 nm. Reprinted with permission from [18]. Copyright 2013 Springer.

While one-photon excited SERS depends on the field amplitude enhancement in the near-field to the power of four (see Equation 2), surface enhanced hyper Raman scattering (SEHRS) increases with the field enhancement factor *A*(ν) to the power of six, because of its quadratic dependence on the excitation intensity [44–45]. This different dependence allows to infer the field enhancement *E*_loc_^2^/*E*_0_^2^ in the hot spots of the nanoaggregates from the ratio between their one- and their two-photon excited surface enhanced Raman and hyper Raman response, respectively. Despite very different signal levels of “normal” Raman and hyper Raman scattering, SERS and SEHRS spectra appear at comparable signal levels and can be measured in the same spectrum as it is demonstrated in Figure 4 [44]. This shows that hyper Raman scattering must experience a much stronger enhancement effect than Raman scattering. Since it is reasonable that the chemical enhancement effect for Raman and hyper Raman scattering is on the same order of magnitude, we can ascribe extremely high SEHRS signals to the near-field. Comparing SEHRS-and SERS-signal levels in more detail [44–45], we find SEHRS enhancement levels that are up to 10^6^ times higher than the SERS enhancement levels. This results in an enhancement on the order of 10^3^ for the field amplitudes in the hottest spots of the near-field (see Equations 2 and Equations 3). Field enhancement factors of 10^3^ give rise to “electromagnetic” SERS enhancement factors on the order of 10^12^. With a “chemical” contribution to SERS enhancement on the order of 10^2^ [42], the total SERS enhancement factors reach 10^14^ as we inferred from anti-Stokes to Stokes signal ratios in SEPARS experiments. An interesting question is the dependence of the near-field enhancement on the photon energy. The ratio between SEHRS and SERS signals measured vs the excitation wavelengths delivers direct information about this dependence. Figure 5 shows the result of experiments, in which a tunable ps Ti:sapphire was used for excitation. The experiments show an increase of the local optical field with increasing wave lengths. Increasing intensities for local fields in the hot spots with decreasing photon energy have also been theoretically predicted [46].

#### Probing the number of hottest hot spots and their geometrical size

Near-field intensities in the vicinity of plasmonic nanostructures such as silver nanoaggregates show strong spatial variations. Very early SERS experiments already did show that an extremely high enhancement level, as it was obtained in SERS vibrational pumping, is available for very few molecules only [20]. Our experiments that were reported in the previous section exploit an extremely high enhancement level as it is available in these hottest spots. This was achieved by working at a very low concentration of the target molecules so that molecules exclusively reside in the hottest hot spots [29]. In the following, we want to determine how many molecules can find a place at the hottest hot spots. Based on this information, we can infer the number of hot spots and their geometrical size.

#### SERS signal vs concentration of the target molecule

**Figure 6:**
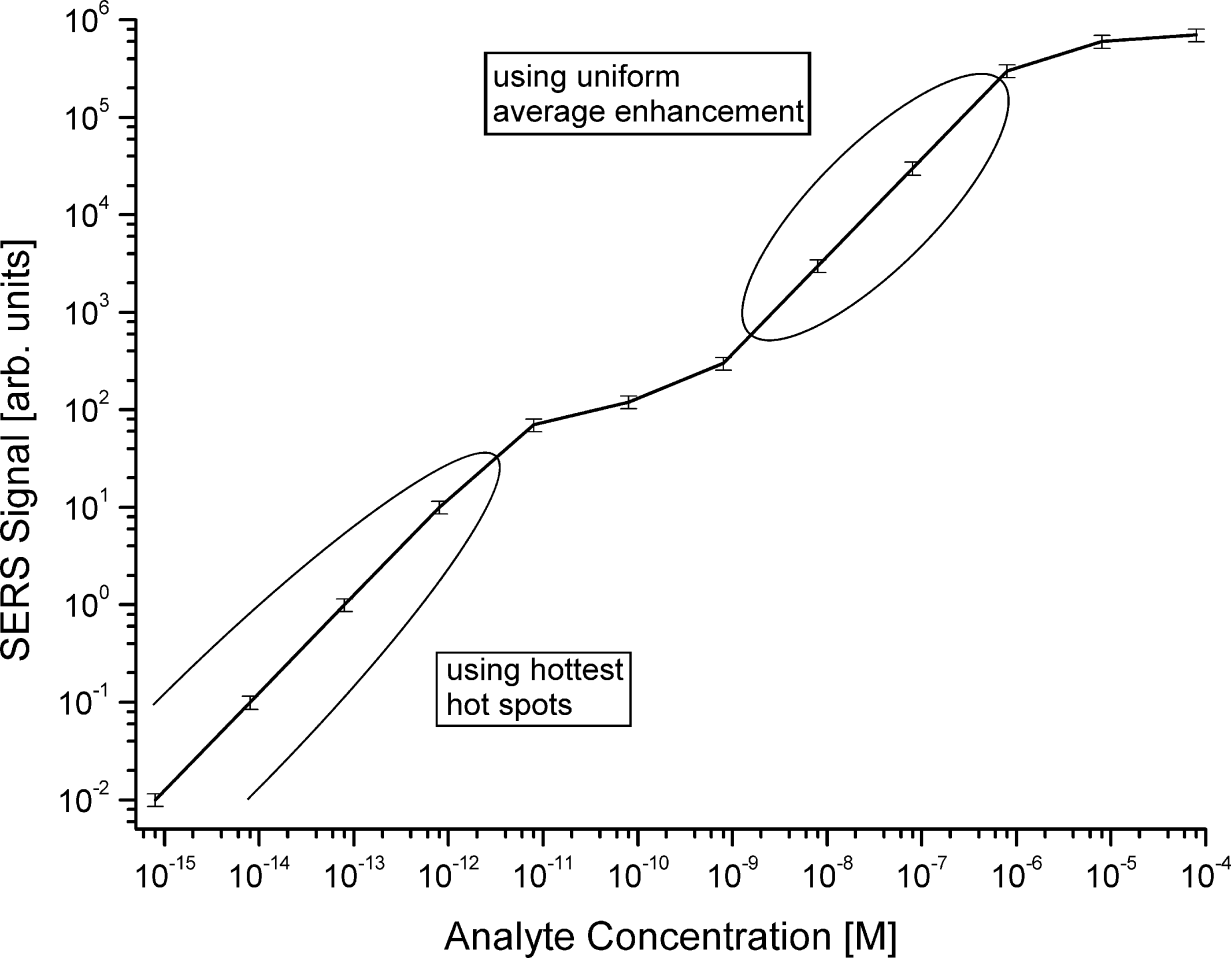
SERS signal of the 1510 cm^−1^ Raman line of rhodamine 6G (see Figure 7) vs concentrations of the molecule between 10^−15^ and 10^−6^ M, excitation at 830 nm.

The experiments were performed in aqueous solutions of silver nanoaggreates described above (Figure 1). The concentration of the nanoaggregates was on the order of 10^−10^ M. A NaCl solution of 10^−3^ M concentration was added to the solution of the aggregates in order to achieve an optimum chemical SERS-enhancement. Each of the silver aggregates provides a total enhancement factor on the order of 10^14^ when 830 nm excitation is used. This level of enhancement was confirmed by checking anti-Stokes to Stokes signal ratios in SEPARS experiments. SERS samples of rhodamine 6G concentrations between 10^−15^ and 10^−6^ M were prepared by adding aqueous solutions of the dye at appropriate concentrations in 1:15 ratios to the solutions of silver nanoaggreates. After non-resonant 830 nm excitation, the Raman scattered light was collected in an 180°-scattering geometry from probed volume of a 10–20 nL inside 1 mL of sample solution by using the same microscope objective for excitation and collection of the scattered light. Figure 6 shows the dependence of SERS signal on the concentrations of the target molecule.

**Figure 7:**
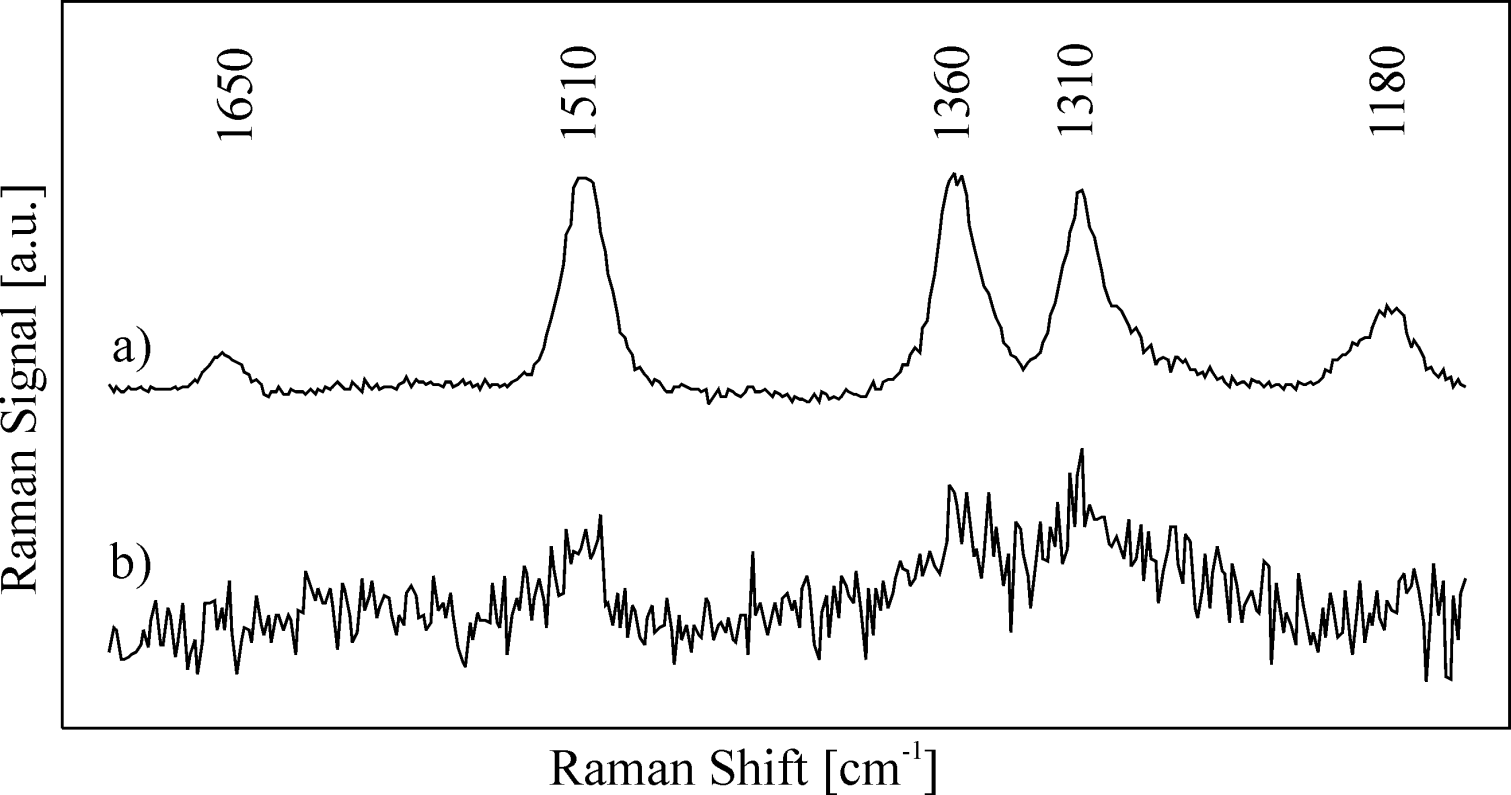
SERS spectra collected from a 10 nL probed volume at concentrations of 10^−15^ and 10^−12^ M of rhodamine 6G in an aqueous solution of silver nanoaggregates, excitation at 830 nm.

For illustration, Figure 7 displays two SERS spectra, which were measured at low concentrations of rhodamine 6G molecules. Due to a relatively large scattering volume of approx. 10 nL, these low-concentration SERS spectra are “many molecule spectra” and were collected from ca. 6 000 (spectrum a) and 6 molecules (spectrum b). The silver nanoaggregates move in and out of the probed volume because of Brownian motion. However, for the applied probed volumes (≥10 nL) the number of analyte-loaded nanoaggregates in the probed volume remains statistically constant also at low analyte concentrations.

Coming back to the plot shown in Figure 6, we can see a linear dependence (slope 1 in the double-logarithmic plot) for analyte concentrations between concentrations of 10^−15^ and 5 × 10^−12^ M, i.e., which are at least two orders of magnitude below the concentration of the nanoaggregates. In this concentration range with *c*_analyte_ << *_C_*_nanoagggregate_, it is statistically unlikely that two molecules are attached to the same aggregate. Each molecule finds its own enhancing nanostructure where it can occupy the hottest hot spots. As we discussed above, high field gradients might direct single molecules to the hottest spots [22,29,38].

Conducting experiments in this low concentration range and reducing the probed volume to a size that only one target molecule or less on average are present in the volume results in a Poisson distribution of the scattering signals measured in time sequence. This statistics reflects the probability of the presence of 0, 1, 2, or 3 molecules in the probed volume during the actual measurement [31–33,47]. Both, the linear increase of the SERS signal vs the concentration of the target molecules in a “many-molecule experiment” displayed in Figure 6 as well as the Poisson distribution of scattering signals in single molecule SERS indicate that each molecule experiences the same extremely high SERS enhancement level, i.e., that molecules reside exclusively in the hottest hot spots.

When the concentration of the target molecule approaches the concentration of the nanoaggregates, the linear relation between the concentration and the SERS signal no longer exists. In this “transition range” statistically more than one molecule can occupy the same nanoaggregate. Experiments show that in the concentration range of the analyte between ca. 10^−11^ and 10^−9^ M, i.e., around a 1:1 concentration of target molecules and nanoaggregates, the SERS signal increases much slower than expected. This indicates that molecules now also start to occupy spots that provide lower enhancement levels at relatively wide intensity distributions. In agreement with losing the linear dependence of SERS power vs analyte concentration at *c*_analyte_ ≈ *c*_nanoagggregate_, single molecule SERS experiments performed in this concentration range also show no Poisson statistics in the distribution of the measured signals.

For higher concentrations of the analyte with *c*_analyte_ >> *c*_nanoagggregate_, concentrations between ca. 10^−9^ and 10^−6^ M in Figure 6, the SERS signals depend again linearly on the analyte concentrations. Under these conditions, 100–10000 molecules are attached to the same nanoaggregate and experience an average uniform enhancement. This regime is used in numerous applications of SERS for quantitative studies, such as in chemical analysis (see examples in [48]).

The loss of the linear relation between the concentration of the target molecule and the SERS signal at approximately 1:1 concentration between aggregates and molecules indicates that only one molecule per nanoaggregate can experience an extremely high enhancement, i.e., there exists only one hottest hot spot per nanoaggregate of about 100 nm dimension (see Figure 1). Moreover, the hottest spot must have very small dimensions since it provides space for only one molecule. Considering a nanoaggregate at a dimension of ca. 100 nm (Figure 1), its surface area is on the order of 3 × 10^−14^ m^2^ and assuming a hot spot at the dimension of a small molecule, i.e., on the order of 10^−20^ m^2^, implies that only 0.00003 % of the surface of the nanoaggregates provide electromagnetic SERS enhancement factors on the order of 10^12^. For more regular Ag films over nanospheres (AgFON) substrates, it has been found that 0.0003% of the surface provides an enhancement factor larger than 10^10^ [49] and 0.003% exhibit enhancement factors larger than 10^9^ [50].

## Conclusion

SERS experiments at the single molecule level open up interesting ways for probing the optical near-field in the hottest hot spots of plasmonic nanostructures. Our studies identify field enhancement factors on the order of 10^3^ with corresponding electromagnetic SERS enhancement factors on the order of 10^12^ for one-photon excited surface enhanced Raman signals and 10^18^ for two-photon excited surface enhanced hyper Raman signals. In agreement with theory [46], the intensities obtained in the hottest spots of the plasmonic near-field increase with increasing wavelengths. Monitoring the SERS signal while increasing the number of target molecules shows that in the near-field of nanoaggregates composed by 4–8 individual silver particles with dimensions of ca. 100 nm (Figure 1) only one hottest hot spot per nanoaggregate exists and that the dimension of these hottest spots must be in the subnanometer range.

Near-fields are always related to high field gradients. Beyond the capabilities for probing the near-field by SERS discussed previously, SERS studies can also provide interesting ways for probing field gradients. Due to the failure of the assumption that the field is constant over molecular dimensions, new selection rules and polarization behavior in Raman signals can be obtained, which could be used to infer information about field gradients [51].
